# Association Among Pelvic Girdle Pain, Diastasis Recti Abdominis, Pubic Symphysis Width, and Pain Catastrophizing: A Matched Case–Control Study

**DOI:** 10.1093/ptj/pzab311

**Published:** 2022-01-05

**Authors:** Małgorzata Starzec-Proserpio, Daria Lipa, Jacek Szymański, Agata Szymańska, Anna Kajdy, Barbara Baranowska

**Affiliations:** Department of Midwifery, Centre of Postgraduate Medical Education, Warsaw, Poland; St. Sophia Specialist Hospital, Warsaw, Poland; First Department of Obstetrics and Gynecology, Centre of Postgraduate Medical Education, Warsaw, Poland; Department of Rehabilitation, Faculty of Medical Sciences, Medical University of Warsaw, Warsaw, Poland; Department of Reproductive Health, Centre of Postgraduate Medical Education, Warsaw, Poland; Department of Midwifery, Centre of Postgraduate Medical Education, Warsaw, Poland

**Keywords:** Diastasis Recti, Pelvic Girdle Pain, Postpartum, Ultrasound

## Abstract

**Objective:**

Pregnancy-related pelvic girdle pain (PGP) may persist or occur postpartum and negatively affects women’s lives. There is uncertainty regarding the association between the structures of the bony pelvis, diastasis recti abdominis (DRA), pain processing, and PGP and to what extent these factors should be considered during physical therapy. This study aimed to evaluate the differences between women with and without PGP shortly after delivery regarding the separation of a pubic symphysis, DRA, and pain catastrophizing.

**Methods:**

Women diagnosed with PGP 24 to 72 hours after vaginal delivery were matched to pain-free controls according to age and parity. Ultrasound evaluations of diastasis recti (interrecti distance [IRD]) during rest and curl-up task and pubic symphysis (interpubic width) were performed. The Pain Catastrophizing Scale was used to assess the level of catastrophizing. A special Cox regression model was used to fit a conditional logistic regression for a 1:2 matched case–control study.

**Results:**

Thirty-five women with clinically diagnosed PGP and 70 matched controls were included in the study. The PGP group had a significantly higher pre-pregnancy body mass index than the control group. After adjusting for body mass index in multiple conditional logistic regression, the interpubic distance (odds ratio = 1.64; 95% CI = 1.22 to 2.20) and IRD during curl-up (odds ratio = 2.01; 95% CI = 1.08 to 3.74) were significantly associated with PGP. Pain catastrophizing and IRD at rest were not associated with PGP in univariable or multivariable analysis.

**Conclusions:**

Pain catastrophizing is similar for women with and without PGP early postpartum. However, the degree of the pubic symphysis and rectus abdominis separation during the curl-up task are positively associated with PGP shortly after delivery.

**Impact:**

This study indicates that a reconsideration of the way we look at DRA is warranted. The development of a more comprehensive assessment including objective measurements and a biopsychosocial understanding is needed to inform directions for further postpartum physical therapy.

## Introduction

Pelvic girdle pain (PGP) is defined as pain located between the posterior iliac crest and the gluteal fold, particularly in the area of the sacroiliac joints and/or the pubic symphysis. It may also radiate to the posterior thigh. Individuals with PGP have difficulties in maintaining a standing or sitting position.^1^ Although the guidelines exclude lumbar causes of pain in the diagnosis of PGP, many pregnant women experience low back pain in addition to pain in the pelvic area.^2^ The pain is usually diagnosed during pregnancy, but it can occur (or become more severe) within 3 weeks after delivery.^3^ It has been estimated that 20% to 25% of pregnant women experience PGP severely enough to seek medical care.^1^^,^^3^ Although the majority recover spontaneously postpartum, it has been reported that 1 in 10 women who experience PGP during pregnancy may experience severe consequences for up to 11 years after delivery.^4^ The consequences of PGP vary, from only minor pain and disability to severe pain, disability, reduced quality of life, and absence from work.^4^^,^^5^ The multifactorial etiology of PGP remains poorly understood, making recovery difficult for many women. A combination of hormonal and biomechanical aspects, inadequate motor control, and stress on ligament structures are the most common hypotheses behind the development of PGP.^1^^,^^6^

In the physical therapy management of PGP, a couple of the important structures are the bony pelvis and the myofascial system of the anterior abdominal wall, mainly the separation of the pubic symphysis and diastasis recti abdominis (DRA). It is essential to distinguish PGP-related pubic symphysis pain from peripartum diastasis of the pubic symphysis, characterized by the separation of the pubic bones above the physiological range (>1 cm^7^). The incidence of the peripartum diastasis of the pubic symphysis ranges from 1:300 to 1:30,000 cases.^8^ When a high rate of peripartum diastasis of the pubic symphysis is reported, it may result from misdiagnosis and the underrecognition of PGP.

DRA is commonly defined as a separation of 2 muscle bellies of the rectus abdominis, which leads to increased interrecti distance (IRD). The widening occurs due to the stretching and thinning of the linea alba due to abdominal expansion and hormonal changes during pregnancy.^9^ The DRA incidence is 27% to 100% among women in late pregnancy and 30% to 68% among postpartum women.^10^^,^^11^ It has been suggested that the muscles and fascia of the lumbopelvic region play a significant role in musculoskeletal function. Greater IRD has been suggested to constitute a nonoptimal myofascial system that fails to achieve optimal strategies for transferring loads through the abdominal canister, including the bony structures, pelvic floor muscles, diaphragm, and abdominal muscles.^12^ It has been demonstrated that pregnancy-related changes in the rectus abdominis are associated with the compromised efficiency of the abdominal muscles to control the movement of the pelvis against resistance.^13^ Although DRA is often suggested to be related to lumbopelvic pain, the research results are inconclusive: some confirm this relationship,^14^^,^^15^ whereas others do not.^16–18^

Recently, more attention has been paid to the role of psychosocial factors in the etiology and pathogenesis of PGP.^19^^,^^20^ Catastrophizing seems to be associated with lumbopelvic pain and postpartum physical ability.^21^ In addition, the tendency for catastrophizing is related to long-term PGP.^4^ An assessment of pain catastrophizing has also been recommended by clinical practice guidelines for PGP.^22^

This study aimed to investigate a potential association between pubic symphysis, IRD, and pain catastrophizing in women with and without clinically diagnosed PGP in the first days postpartum.

## Material and Methods

### Study Design

The study was designed as a 1:2 matched case-control study. Each woman with PGP was matched to 2 women without PGP by age (±5 years) and the number of previous deliveries. The study was approved by the Bioethics Committee of the Centre of Postgraduate Medical Education in Warsaw, Poland (KBE/10B/2018), and was prospectively registered at https://clinicaltrials.gov/ (NCT03835650). The study participants were recruited between April 2019 and December 2020 in St Sophia Specialist Hospital in Warsaw, Poland, among women routinely examined by physical therapists between 24 and 72 hours postpartum. Physical therapists screened those reporting symptoms of PGP against the recommended criteria.^1^ All of the participants spoke and understood Polish.

### Participants

The cases included were women between 18 and 45 years old, after vaginal delivery, and reporting pain in the pelvic girdle region, confirmed by indicating their pain on a body chart. The examination was performed by 2 experienced women’s health physical therapists trained in the musculoskeletal ultrasound assessment. Both physical therapists attended a mandatory half-day training together, reviewing the organizational aspects of the study and the clinical examination procedures. The training aimed to promote consistency and avoid bias throughout the data collection process.

For PGP classification, existing guidelines and previous reports were used.^1^^,^^4^^,^^23^^,^^24^ The examination consisted of functional tests dedicated to pelvic girdle assessment. At least 2 tests had to be positive to allocate the participant in the PGP group: Posterior Pelvic Pain Provocation test, distraction test, compression test, palpation of the pubic symphysis, modified Trendelenburg test, and active straight leg raise test (ASLR). Two tests were used for pubic symphysis pain because the pubic symphysis pressure test has been shown to be false positive in women with no PGP.^25^ If 2 tests for pubic symphysis were positive, 1 more positive test was required for the allocation to the PGP group. This allowed for more reliable results in the diagnosis of PGP during the first days after vaginal delivery, when the pubic symphysis and perineal area are compromised*.* This was done to increase the certainty we were including women presenting with “real” PGP only. For the ASLR test, the scores on both sides were added, and the total score ranged from 0 to 10. An ASLR total score of 4 and more was considered positive.^24^^,^^26^ When assessing the pain provocation tests, it was recorded if a familiar pain was provoked. Additionally, the participant was screened for end-range movements in the lumbar region (flexion, extension, lateral flexion, and rotation) to exclude lumbar causes of pain experienced in the pelvic girdle region.

The control group consisted of women recruited in the same way, without complaints of pain in the pelvic girdle region. They were shown a graphic with marked PGP locations and were asked if they experienced pain in that area, especially during walking, standing, or rolling to the side in the bed. If the answer was “no,” they were included in the study. Apart from the presence of pain, all the remaining inclusion criteria were the same as for the PGP group. The exclusion criteria for both groups were (1) severe postpartum complications (internal bleeding, femoral artery embolism, and pelvic fracture); and (2) other diseases that may mimic PGP (rheumatoid arthritis, ankylosing spondylitis, Scheuermann disease, Ehlers-Danlos syndrome, spinal surgeries, nerve root compression, and spondylolisthesis) and a positive neurological examination (nerve-root compression assessed by Lasegue test and sensory deficits).

### Variables/Outcome Measures

The severity of the symptoms related to PGP was assessed using the Visual Analogue Scale^27^ and the Polish version of the Pelvic Girdle Questionnaire.^28^^,^^29^

**Figure 1:**
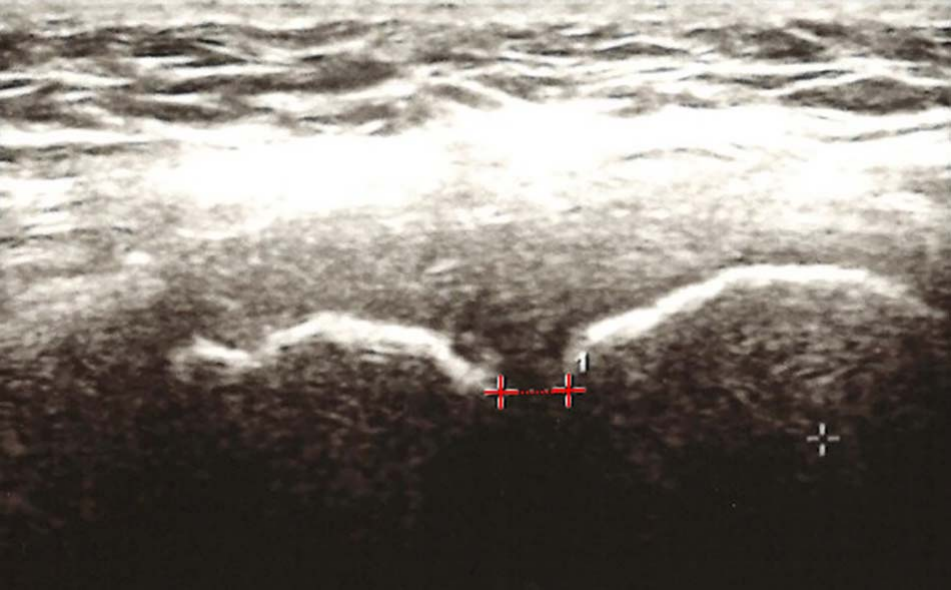
Interpubic width measurement using ultrasound imaging. The line from which the distance was measured is indicated by a dotted line.

Pubic symphysis width was assessed by ultrasound using a linear array transducer with the individual in a supine position. The transducer was applied in the transverse plane in the suprasymphysis area and moved caudally until the bone structures were detected. Then, the probe was tipped over at angles between 30 and 45 degrees in the transverse plane to reach the best view of the pubic symphysis.^30^ The interpubic distance was measured with electronic calipers in the narrowest space.^7^^,^^31^ Ultrasonographic measurements of pubic symphysis diastasis (Fig. 1) are faster, safer, and more accessible than X-ray imaging. This method of measuring symphysis width has been used previously and demonstrated good comparability with the radiographic method^32^^,^^33^ and showed a correlation coefficient of 0.850 (when measured by 1 radiologist and 1 ultrasonographer).^33^

The diastasis recti was measured by palpation and ultrasound. The assessments were performed 2 cm over the umbilicus.^34^^,^^35^ The participants were positioned supine, and the desired measurement location was marked with a water-soluble pen. The palpation examination procedure was taken from previous reports.^34^^,^^35^ The women were asked to perform an abdominal curl-up by raising their head and upper torso until their shoulder blades left the examination bed. The physical therapist placed her fingers across the linea alba at the level of the marked point so that the width of her fingers would fill the gap between the edges of the abdominal rectus muscles. The reported interrater reliability of measurements obtained by palpation was 0.702 (when measured by Spearman’s rho) and 0.534 (when weighted kappa was used).^34^ To mitigate the measurement bias between the 2 examiners, the number of fingers filling the gap between the rectus abdominis muscles was measured by an electronic caliper. Additionally, the stiffness and distortion of the linea alba were assessed. This was defined as the “linea alba stability” (stable/distorted). The bulging or collapsing of the abdominal surface in the projection of the linea alba was considered “distorted.”

Ultrasound measurements were taken at the same point in the 2 conditions (abdominal muscles at rest and contracted). The transducer was placed perpendicularly to the abdominal surface at the point corresponding to the skin marker. The medial borders of both the rectus abdominis muscles were visualized. The measuring feature was used to measure the inter recti distance (IRD) by first capturing the image, followed by the examiner determining the location of the medial borders of the rectus abdominis muscles and using the onscreen cursor to mark the distance between the right and left muscle bellies. Additionally, attention was paid to the pressure imposed on the probe to avoid reflexive responses from the muscles and the participant. This procedure was also used in the previous studies where IRD was measured.^34^^,^^36^^,^^37^ All the participants were blinded to their measured IRD.

**Figure 2:**
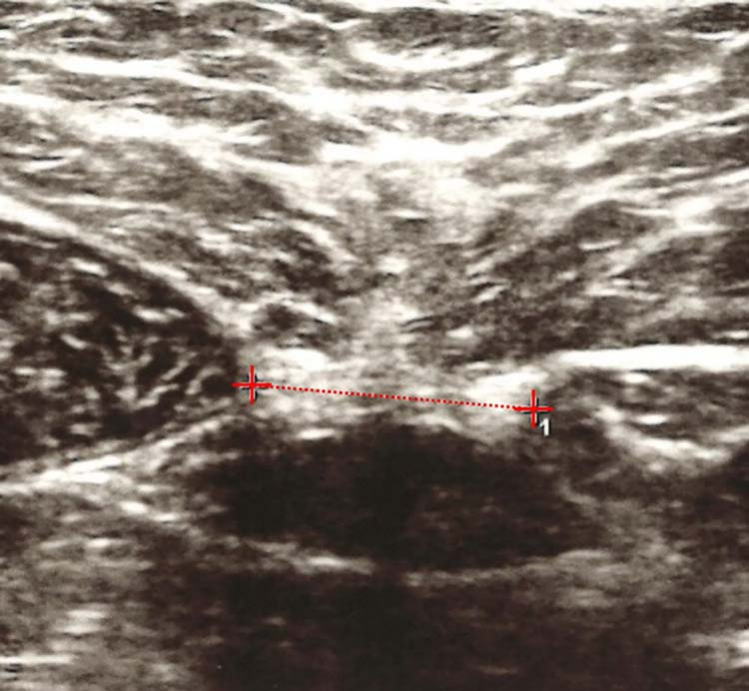
Interrecti distance (IRD) measurement using ultrasound imaging. The line from which the IRD was measured is indicated by a dotted line.

Ultrasonography was an accurate method to measure the IRD above the umbilicus compared with a surgical compass during an abdominoplasty.^38^ A good to very good reliability for IRD measurements with this method was reported.^36^ When using ultrasound imaging to measure IRD in women (Fig. 2), it is acceptable for different therapists to compare IRDs between individuals if IRD is measured above or below the umbilicus (intraclass correlation coefficients ranged from 0.63 to 0.96).^39^

All the ultrasound measurements were performed using the Voluson P6 (GE Healthcare Systems; Chicago, IL, USA) ultrasound with a 37-mm linear transducer (4–12 MHz). The palpation examination served to fill in for the missing IRD measurements if the diastasis was wider than the linear transducer.

The Pain Catastrophizing Scale (PCS)^40^ was used to assess the mental processing associated with experiencing pain. This questionnaire is one of the patient-reported outcomes indicated for use with individuals with PGP.^22^ The scale contains 13 statements describing various thoughts and feelings that may be related to pain. Each item is rated on a Likert scale, ranging from 0 (not at all) to 4 (all the time). Higher scores indicate a higher level of catastrophizing. All the participants filled it out after the physical examination.

### Statistical Analysis

Sample size calculation was based on the previous report on the sonographic assessment of the symphyseal joint with reference to pain in the pelvic region.^41^ With 95% power, an allocation ratio of 2, and for a 5% significance level, at least 50 participants (17 in the PGP and 33 in the control group) should be included (G*Power 3.1; Heinrich-Heine-Universität Düsseldorf, Germany).^42^ Considering the possibility of missing data, we included 105 participants (35 in the study and 70 in the control group). Means and SD were calculated for continuous variables. Categorical data are presented as counts and percentages. The differences between the cases and the controls were analyzed with the Student *t* test for normally distributed continuous variables and the Mann–Whitney U test for data that were not normally distributed. Spearman rho was used to measure the strength of the relationship between the palpation examination and the ultrasound as well as for the correlations between pain severity and ultrasound measurements. A special Cox regression model was used to fit a conditional logistic regression procedure for 1:2 matched case-control studies. The results are given as odds ratios (OR) with 95% CI. Due to the design of the study, all the analyses were adjusted for age and parity. Missing data were not included in the analysis. The outcomes were considered statistically significant when *P* < .05. Statistical analyses were performed using SPSS software, version 27 (IBM, Armonk, NY, USA).

### Role of the Funding Source

The funder played no role in the design, conduct, or reporting of this study. No project funding sources had a role in the data collection or the analysis, and the results did not require funding source approval prior to publication.

## Results

Thirty-five women with clinically confirmed PGP were successfully matched to 70 controls according to age and parity. There were difficulties in matching, where 1 individual with PGP delivered vaginally after previous 3 cesarean sections. She was matched to 2 other participants after 4 labors with a history of 1 and 2 cesarean sections. Both groups did not differ in age, parity, weight gain during pregnancy, and education. However, body mass index (BMI) before pregnancy was statistically higher in the PGP group compared with the controls. The characteristics of the study groups are presented in Table 1. As shown in Table 2, women with PGP were afflicted by pain and functional limitations.

**Table 1:** Differences Between Cases With Pelvic Girdle Pain and Controls

**Characteristics**	**Cases (n = 35)**	**Controls** **(n = 70)**	***P***
Age, mean (SD), y*^a^*	32.86 (4.51)	33.00 (4.22)	.873
Parity, mean (SD)	1.77 (0.89)	1.77 (0.89)	.997
Body mass index before pregnancy, mean (SD), kg/m^2^	24.52 (4.55)	22.52 (3.18)	**.037**
Weight gain during pregnancy, mean (SD), g	14.29 (4.82)	15.11 (4.85)	.274
Infant body mass, mean (SD), g	3460.14 (431.80)	3454.71 (414.47)	.743
Higher education, n (%)	31 (88.6)	65 (92.9)	.460
Pelvic girdle pain during pregnancy (self-reported), n (%)	25 (71.4)	28 (40)	**.002**
Duration of 2nd stage of labor, mean (SD), min	29.57 (25.05)	27.44 (21.35)	.922

a Normally distributed data, tested with the Student *t* test. All the other continuous variables were not normally distributed and were tested with the Mann–Whitney U test. Values in bold are results that reached statistical significance.

**Table 2:** Characteristics of Cases With Pelvic Girdle Pain (n = 35)

**Characteristics**	**Mean (SD)**	**Number (%)**
Pelvic Girdle Questionnaire (0–100%)	59.57 (14.51)	
Visual Analogue Scale (0–100 mm)	56.88 (13.78)	
No. of positive functional tests	5.26 (2.42)	
Type of pelvic girdle pain		
Posterior pelvic pain		7 (20)
Unilateral posterior pain		9 (25.7)
Symphyseal pain		7 (20)
Pelvic girdle syndrome (all 3 pelvic joints)		7 (20)
Unilateral posterior pain + symphyseal pain		5 (14.3)

The results of the palpation examination showed a good correlation with the ultrasound measurements (*r*^2^ = 0.71), and the differences between them were not statistically significant (*P* = .33). This allowed the missing ultrasound values to be filled in where the IRD exceeded the transducer length.

There were no statistically significant differences between the groups regarding the IRD ultrasound measurements, pain catastrophizing, and linea alba stability. However, univariable analysis showed that women with PGP had statistically significantly bigger pubic symphysis width compared with the controls (*P* ≤ .001) (Tab. 3). The additional analysis of the PGP group did not reveal any statistically significant correlation between the pubic symphysis width, the IRD, and either the pain severity (Visual Analogue Scale) or the Pelvic Girdle Questionnaire scores (*P* > .05).

**Table 3:** Differences Between Cases With Pelvic Girdle Pain (N = 35) and the Controls (N = 70)^a^

**Measured Variables**	**Cases**	**Controls**	***P***
Pubic symphysis width, mean (SD), cm	0.84 (0.38)	0.61 (0.19)	**.001**
IRD at rest, mean (SD), cm	2.83 (0.98)	3.03 (0.71)	.405
IRD during curl-up, mean (SD), cm	2.73 (1.84)	2.49 (1.12)	.786
Linea alba stability			
Stable, n (%)	27 (77.1)	52 (74.3)	.749
Distorted, n (%)	8 (22.9)	18 (25.7)	
Pain Catastrophizing Scale (0–52), mean (SD)	17.14 (8.80)	16.17 (9.50)	.386

^a^ Not normally distributed data, tested with the Mann–Whitney U test. IRD = interrecti distance. Values in bold are results that reached statistical significance.

The results of multivariable regression analysis, adjusted for BMI before pregnancy, are presented in Table 4. The pubic symphysis width (*P* ≤ .001) and the IRD during the curl-up task (*P* = .03) were significantly associated with PGP. In our sample, women with wider pubic symphysis and wider IRD were more likely to have PGP.

**Table 4:** ORs of Possible Factors Associated With Pelvic Girdle Pain Using Conditional Multiple Regression^a^*^,^**^b^*

	**Adjusted OR (95% CI)**	***P***
Pubic symphysis width, cm	1.64 (1.22 to 2.20)	**.001**
IRD at rest, cm	0.50 (0.22 to 1.14)	.100
IRD during curl-up, cm	2.01 (1.08 to 3.74)	**.028**
Distorted linea alba	0.22 (0.04 to 1.30)	.095
Pain Catastrophizing Scale	1.03 (0.97 to 1.10)	.388

^a^ IRD = interrecti distance; OR = odds ratio.

^b^ ORs are adjusted for body mass index. Values in bold are results that reached statistical significance.

## Discussion

The presented 1:2 matched case–control study showed that women with PGP early postpartum did not present with higher pain catastrophizing and more distortion at the level of linea alba compared with the controls. However, the pubic symphysis width and the IRD during the curl-up task were significantly associated with PGP.

It has been hypothesized that DRA and linea alba dysfunctions are associated with pain in the lumbopelvic area.^9^^,^^10^^,^^12^^,^^14^^,^^43^ However, the available evidence is conflicting. To our knowledge, only 2 authors have found some associations between pain in the lumbopelvic area and the separation of the rectus muscles. Dalal et al^14^ reported a moderate positive and statistically significant correlation between DRA and lumbopelvic pain using the finger width method. Parker et al^15^ found, using a dial caliper, significantly greater amounts of abdominal/pelvic pain in women with DRA than in those without DRA. However, other studies^16^^,^^17^^,^^44^ did not find such associations, and a recent systematic review could not conclude that DRA presence is associated with lumbopelvic pain.^45^ In our study, early postpartum IRD during curl-up task was associated with PGP only in multivariable analysis, after controlling for BMI. It is possible that lack of differences between groups in univariable analysis may be compounded by the baseline differences in pre-pregnancy BMI.

Several factors make our study different from those previously reported. In all the reports mentioned above, DRA was classified using arbitrary cut-off points. However, it is not clear whether the current practice of classifying DRA as an IRD equal or wider than 2 to 2.5 cm/2 fingerbreadths is clinically meaningful, especially because the amount of the separation present (IRD) varies significantly among women.^46^ Because the separation of the rectus abdominis muscles bellies is highly prevalent in the early postpartum period,^11^ we decided not to use arbitrary cut-off points. Additionally, some previous studies did not investigate PGP exclusively and analyzed it grouped with other pain conditions, such as abdominal pain,^15^ as a lumbopelvic pain entity^17^ or pelvic pain.^15^^,^^18^^,^^47^ There is a body of evidence demonstrating that PGP is a distinct condition from low back pain and pelvic pain and, therefore, should be studied separately.^1^

Keshwani et al^18^ investigated whether the size of the IRD measured by ultrasound correlated with pelvic pain severity and did not find an association which is consistent with our results. It may be possible that the separation of the muscle bellies is related to the presence of PGP but not to its severity. Other factors than just the IRD size were suggested to moderate this relationship. Lee and Hodges^48^ demonstrated that some women with DRA present with the capacity of the linea alba to transmit forces independently of the IRD size. Therefore, it was suspected that the women who present with a reduced ability to transmit forces through the linea alba may be more prone to experiencing pain or dysfunction in the lumbopelvic area. Our study did not confirm this association. However, we used a non-validated method to assess the linea alba, only observing its bulging/collapsing. Some more advanced ultrasound imaging techniques for the assessment of linea alba distortion and stiffness are being proposed.^49^^,^^50^

Research studies investigating the associations between PGP and pubic symphysis width presented similar results as in the case of the IRD and are consistent with our results; increased pubic symphysis width may be associated with the presence of pain,^51^ but it was not directly related to pain and symptom severity.^41^^,^^52^ Additionally, pain relief was not accompanied by a resolution of a symphysis separation.^52^ Many clinicians use the term “postpartum symphysis separation” as a clinical diagnosis of pain in the pubic symphysis area. However, only 9 women in our study group and, interestingly, 2 women in the control group met the criteria of pubic symphysis diastasis. Bjorklund et al^53^ and Rustamova et al^54^ measured pubic symphysis intrapartum and presented widened symphyseal joint results in asymptomatic women. This may indicate that increased pubic symphysis width does not have to be symptomatic during the peripartum period.

An outcome measuring tool such as the PCS is essential to aid clinicians in assessing the mental processing associated with pregnancy-related PGP. Clinical practice guidelines for assessment and treatment of PGP have recommended the significance of individuals’ beliefs and perceptions about their pain, measured using this questionnaire.^22^ In our study, pain catastrophizing was not associated with pain. This is contrary to the results of Elden et al,^4^ who studied the factors associated with persistent PGP up to a decade after delivery. In their study, women experiencing PGP had significantly higher levels of pain catastrophizing than women with no PGP when measured using the PCS. Different results may be due to the differences between the studied groups. Elden et al^4^ investigated pain catastrophizing in women with persistent, long-term PGP, whereas we studied women in the early postpartum period with a shorter pain duration.

Additionally, a physical therapist consulted all the women in our study, which was the standard postpartum care in the study setting. Those with pain were reassured about their condition by receiving education about the nature of PGP. Additionally, they were informed when and where they should seek further physical therapy. It was previously shown that short pain neurophysiology education might have the potential to reduce pain catastrophizing.^55^ Although the physical therapy consult was not structured into proper therapeutic pain neuroscience education, we believe it could lower women’s level of pain catastrophizing.

### Strengths of the Study

To our knowledge, this is the first study that examined the relationship between IRD and PGP in a specifically selected group of women with PGP classified according to existing guidelines for PGP and confirmed by clinical examination. Additionally to the robust matching design, we used ultrasound as the only validated and reliable outcome to measure IRD.^56^

### Limitations

A major limitation of this study relates to the examinations provided by 2 separate physical therapists, which could lead to assessor bias. Despite efforts to standardize the data collection procedures, we acknowledge that associated imprecision may have occurred. Another limitation relates to non-validated linea alba stability**/**distortion measurements. Currently, we do not have a full understanding of the linea alba properties and their role in PGP. However, the used form of measurement aimed to inform future studies about the eventual need for further investigation. Finally, due to the observational study design applied, the PGP and measured variables established here are only associations. Insight related to the causality of the studied factors as related to the PGP remains an important issue that requires further investigation.

## Conclusions

This study aimed to evaluate the differences between women with and without PGP shortly after delivery regarding the separation of a pubic symphysis, the IRD, and pain catastrophizing. The results showed that pain catastrophizing is similar for women with and without PGP early postpartum. However, the degree of the pubic symphysis and rectus abdominis separation during the curl-up task shortly after delivery were positively associated with PGP. This could inform directions for further postpartum physical therapy. However, the results of this study cannot be generalized to all women with postpartum PGP. Women with persistent PGP sometime after delivery may experience cumulative effects that lead to pain or dysfunction. This may not be evident in the early postpartum period. Therefore, further studies investigating the abdominal wall, pelvic girdle function, and psychosocial factors—specifically in women with postpartum PGP—are needed to see a trend in changes over time. Adding ultrasound measurements to the available functional tests and pain scales may improve the objectivity and accuracy of the obtained results.

## Clinical Trial

This study was prospectively registered at https://clinicaltrials.gov/ (NCT03835650).
